# Appendix-preserving elective herniorrhaphy for de Garengeot hernia: two case reports

**DOI:** 10.1186/s40792-021-01329-x

**Published:** 2021-11-22

**Authors:** Hiromitsu Imataki, Hideo Miyake, Hidemasa Nagai, Yuichiro Yoshioka, Koji Shibata, Yuichi Kambara, Norihiro Yuasa

**Affiliations:** Department of Gastrointestinal Surgery, Japanese Red Cross Aichi Medical Center Nagoya Daiichi Hospital, 3-35 Michishita-cho, Nakamura-ku, Nagoya, 453-8511 Japan

**Keywords:** De Garengeot hernia, Ultrasonography, Appendicitis, Elective surgery

## Abstract

**Background:**

Emergency appendectomy is often performed for de Garengeot hernia. However, in some cases, there may be a chance to perform an appendix-preserving elective surgery.

**Case description:**

A 76-year-old woman presented to our hospital with complaints of a right inguinal swelling, which we diagnosed as a de Garengeot hernia using computed tomography (CT). B-mode ultrasonography (US) of the mass showed an appendix 4–6 mm in diameter with a clear wall structure; color Doppler US showed pulsatile blood flow signal in the appendiceal wall. Twenty-eight days later, herniorrhaphy with transabdominal preperitoneal repair (TAPP) was performed without appendectomy. Another 70-year-old woman presented to our hospital with complaints of a painful bulge in the right inguinal region. The diagnosis of de Garengeot hernia was made using CT. B-mode US showed an appendix 5 mm in diameter with a clear wall structure. Color Doppler US showed a pulsatile blood signal in the appendiceal wall. Seven days later, herniorrhaphy with TAPP was performed without appendectomy.

**Conclusion:**

De Garengeot hernia is often associated with appendicitis; however, an appendix-preserving elective herniorrhaphy can be performed if US and intraoperative findings do not suggest appendicitis or circulatory compromise in the appendix.

**Supplementary Information:**

The online version contains supplementary material available at 10.1186/s40792-021-01329-x.

## Background

De Garengeot hernia is a femoral hernia that contains the appendix [1]; its incidence has been reported to be 0.15–5% of all femoral hernias [2–4]. Diagnosis is often difficult because of its rarity. Because de Garengeot hernia is often associated with appendicitis or circulatory compromise of the appendix, most surgeons perform emergent herniorrhaphy with appendectomy [5–7]; however, there may be a chance to perform an appendix-preserving elective surgery in certain situations. We report two cases of de Garengeot hernia that were preoperatively diagnosed and treated with elective herniorrhaphy without appendectomy. We have also highlighted the usefulness of ultrasonography (US) in the evaluation of inflammation and circulatory status of the appendix.

## Case presentation

### Case 1

**Fig. 1:**
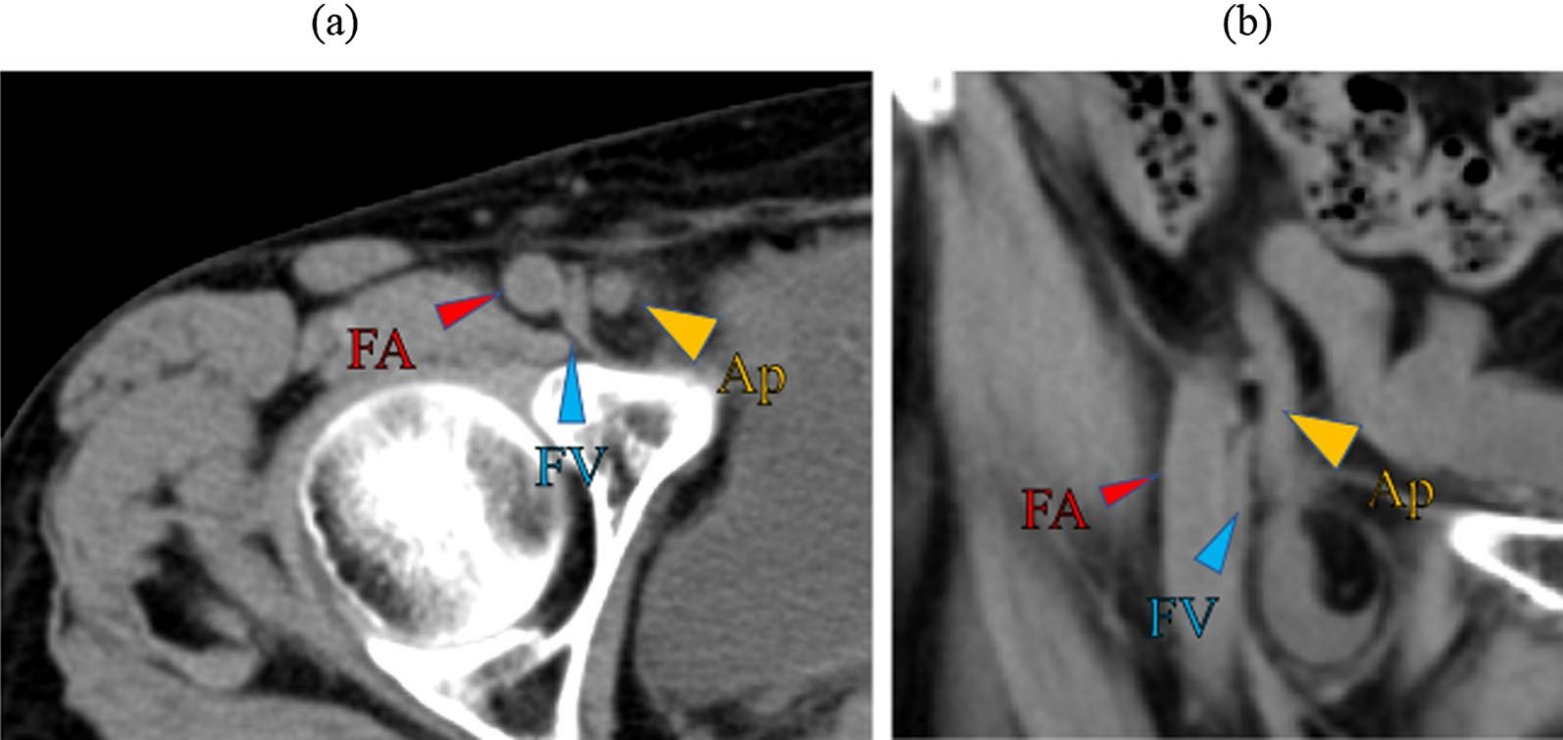
Computed tomography (Case 1). **a** Axial image showing a well-defined isodense structure (Ap) on the medial side of the right femoral vein (FV). **b** Coronal image showing an isodense blind-ended tubular structure (Ap) on the medial side of the right FV protruding from the abdominal cavity. *Ap* appendix, *FA* femoral artery

**Fig. 2:**
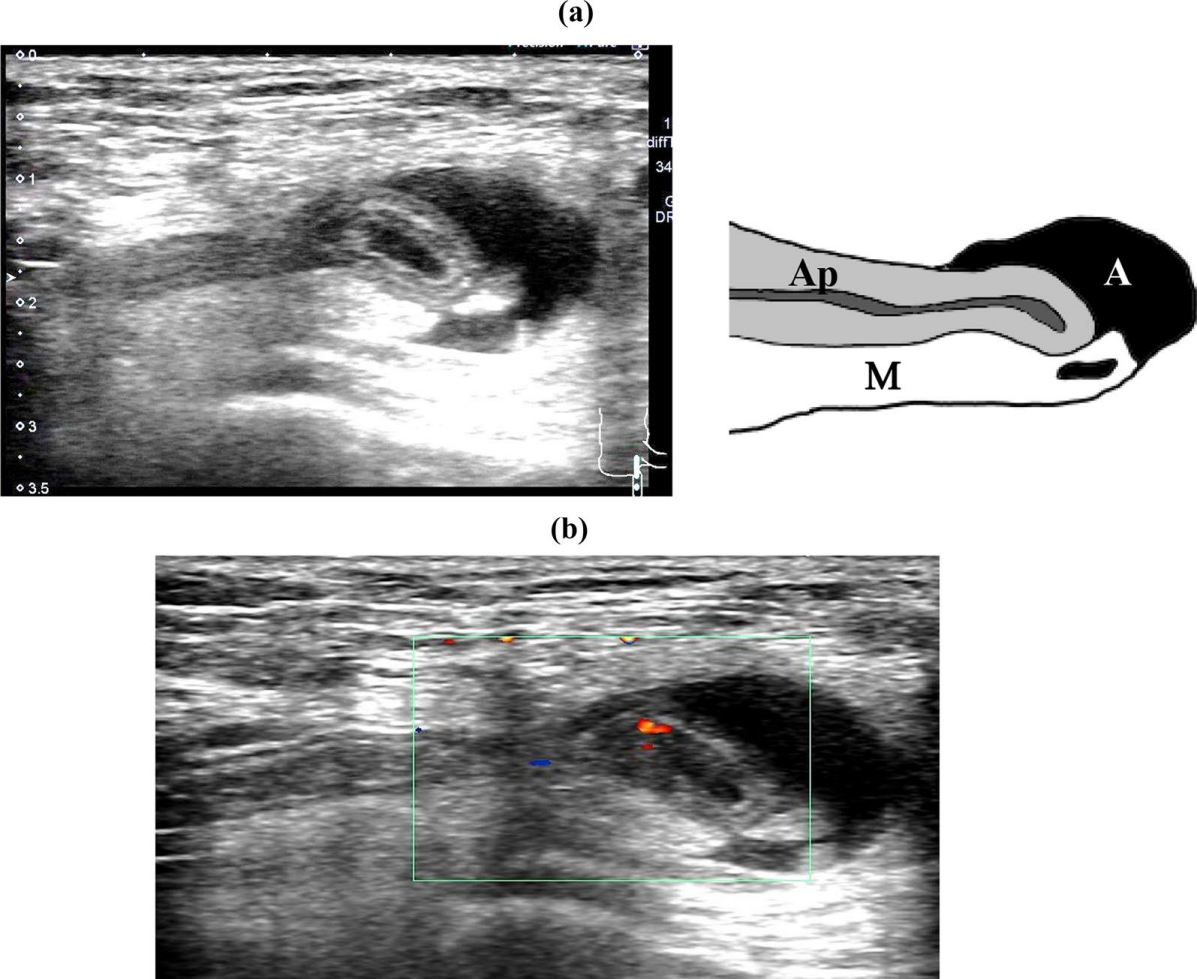
Ultrasonography (US) (Case 1). **a** B-mode US showing a blind-ended hyperechoic luminal structure with five layers extending from the abdominal cavity (diameter: 4 mm at the body, 6 mm at the tip), a reticular hyperechoic area, and a hypoechoic area on the medial side of the right femoral vein. They were diagnosed as appendix (Ap), mesoappendix (M), and ascites (A), respectively. **b** Color doppler US showing pulsatile blood flow signals in the appendiceal wall

A 76-year-old woman presented to our hospital with complaints of a right inguinal swelling. Her body temperature was 35.9 ℃, and the mass was not reducible. Blood tests showed a white blood cell (WBC) count of 3400/mm^3^, hemoglobin 11.9 g/dL, C-reactive protein (CRP) 0.02 mg/dL, albumin 3.7 g/dL, total bilirubin 0.7 mg/dL, blood urea nitrogen 16 mg/dL, and creatinine 0.76 mg/dL, which were not suggestive of an inflammatory reaction. Plain computed tomography (CT) (Fig. 1) revealed a well-defined, isodense, blind-ended tubular structure medial to the right femoral vein. B-mode ultrasonography (US) showed a blind-ended hyperechoic luminal structure protruding from the abdominal cavity (diameter: 4 mm at the body, 6 mm at the tip), a reticular hyperechoic area, and an anechoic area medial to the right femoral vein, which were determined to be the appendix, mesoappendix, and ascites, respectively (Fig. 2a). The appendiceal wall structure (five layers) was clearly visible. Color Doppler US showed pulsatile blood flow signals in the appendiceal wall (Fig. 2b, Additional file 1: video S1). CT and US indicated de Garengeot hernia; however, results of blood studies and US did not suggest appendicitis or appendiceal circulatory compromise. We planned an elective herniorrhaphy, which was performed using a transabdominal preperitoneal approach (TAPP), 28 days later.

**Fig. 3:**
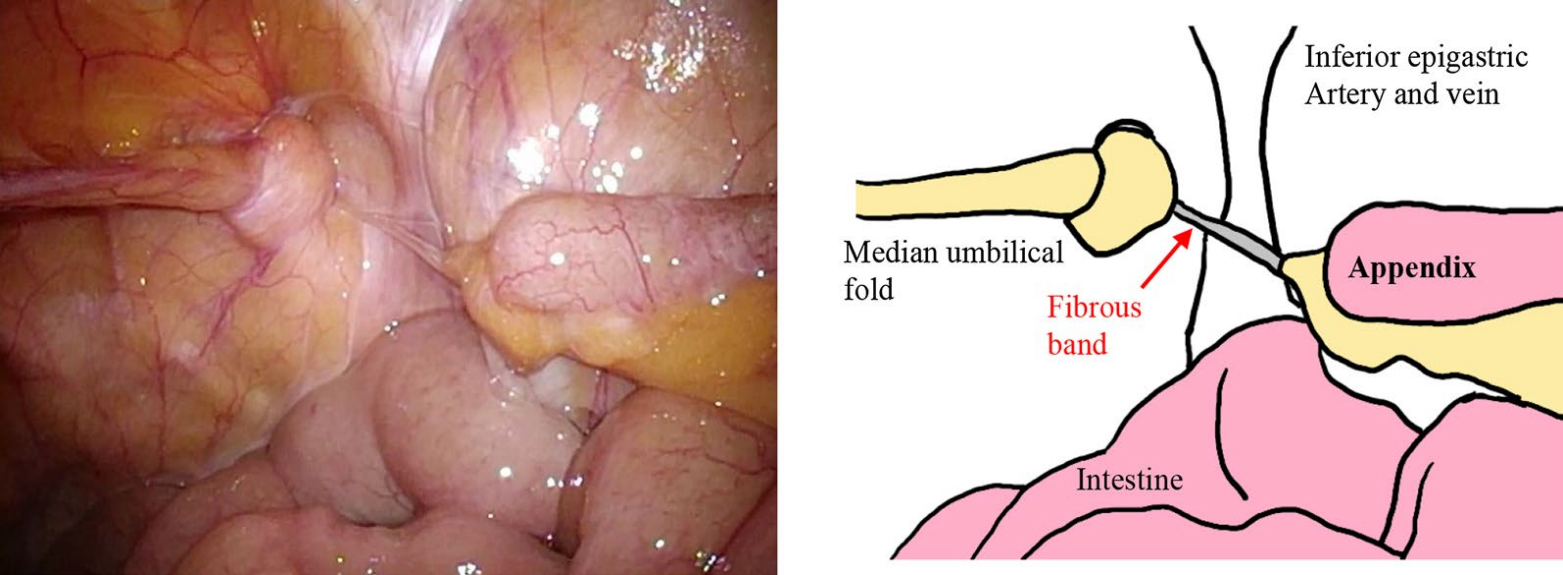
Laparoscopic image (Case 1). The median umbilical fold was incarcerated into the right femoral ring, and the appendix was present in the free abdominal cavity. There was no enlargement or color change in the appendix. A fibrous band was found between the right femoral ring and the appendiceal tip

The patient was placed in the supine position under general anesthesia, and one 5-mm port each was placed on the umbilicus, umbilical level on the right side of the abdomen, and left lower abdomen. Laparoscopy showed incarceration of the median umbilical fold into the right femoral ring and the free appendix in the abdominal cavity (Fig. 3, Additional file 2: video S2). A fibrous band was also found between the right femoral ring and appendiceal tip, suggesting that the appendiceal tip had previously been in the femoral ring. There was no enlargement or color change in the appendix; therefore, appendectomy was not performed. The median umbilical fold was restored to the abdominal cavity, the peritoneum was incised, and parietalization was performed. Versatex mesh (Covidien) 14 cm × 9 cm was placed in the preperitoneal space and fixed with an Absorber Tack 5 mm (Covidien). The peritoneum was closed using continuous suturing with a 3–0 Polysorb (Covidien).

She was discharged 2 days after the surgery and has shown no sign of hernia recurrence or appendicitis during the 6 months that have passed since the surgery.

### Case 2

**Fig. 4:**
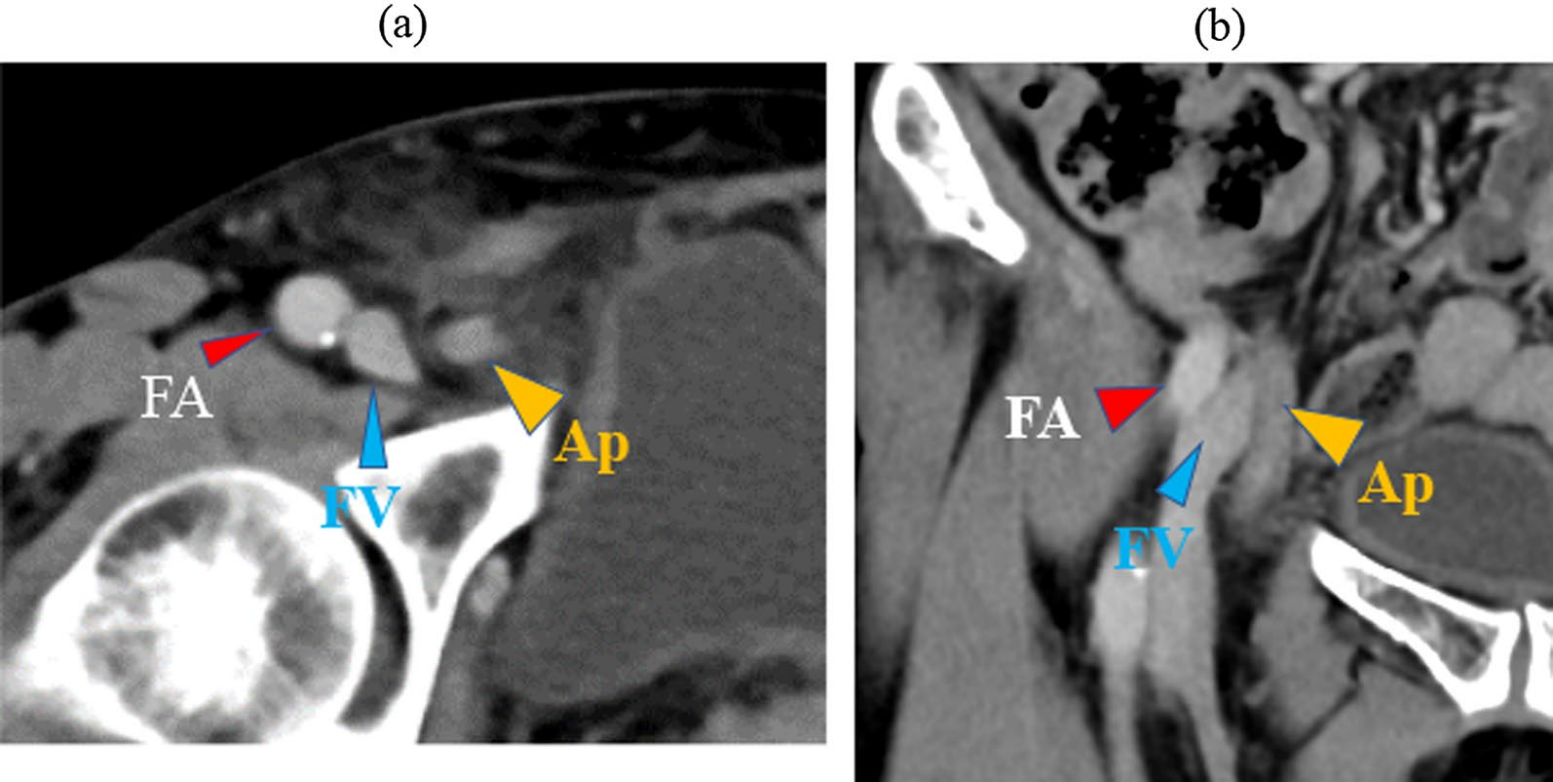
Computed tomography (Case 2). **a** Axial image showing a well-defined structure 6 mm in diameter (Ap) with contrast enhancement on the medial side of the right femoral vein (FV). **b** Coronal image showing a tubular structure continuous with the cecum. *Ap* appendix, *FA* femoral artery

**Fig. 5:**
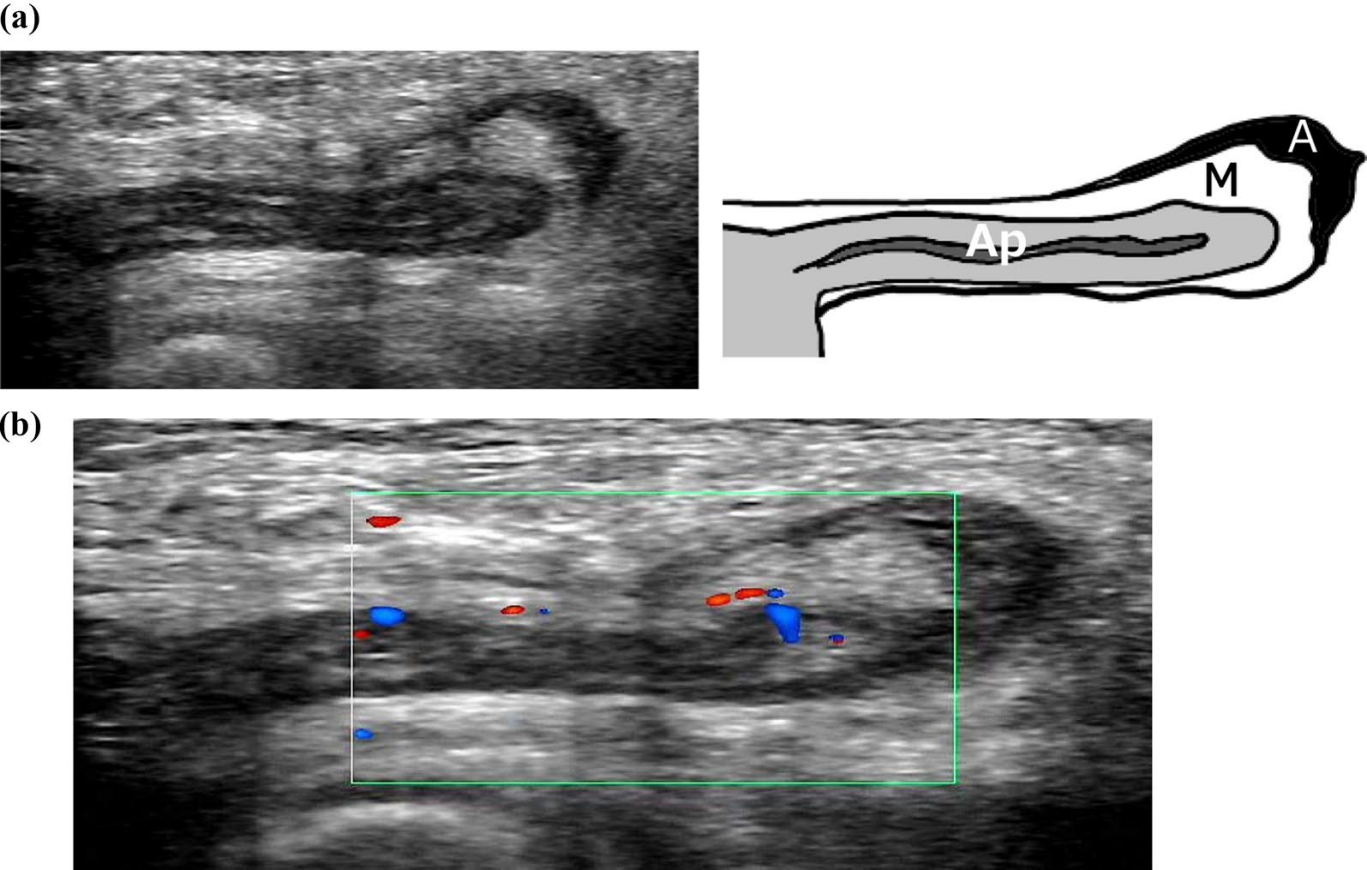
Ultrasonography (US) (Case 2). **a** B-mode US showing a blind-ended isoechoic tubular structure (5 mm in diameter) continuous with the cecum, a surrounding reticular hyperechoic area, and an anechoic area on the medial side of the right femoral vein. They were diagnosed as appendix (Ap), mesoappendix (M), and ascites (A), respectively. **b** Color doppler US showing pulsatile blood flow signals in the appendiceal wall

A 70-year-old woman presented to our hospital with complaints of right inguinal pain and swelling. A 3-cm inguinal mass was palpable; but not manually reducible. Blood test showed slightly elevated WBC count and CRP level (WBC 9500/mm^3^, CRP 2.23 mg/dL). Contrast-enhanced CT (Fig. 4) showed a blind-ended tubular structure, 6 mm in diameter and continuous with the cecum with contrast enhancement medial to the right femoral vein, suggesting that it was the appendix. B-mode US showed a blind-ended isoechoic structure (5 mm in diameter) which was continuous with the cecum, a surrounding reticular hyperechoic area, and an anechoic area medial to the right femoral vein, which were diagnosed as the appendix, mesoappendix, and ascites, respectively (Fig. 5a). B-mode US showed a clear appendiceal wall structure, and color Doppler US showed pulsatile blood flow signals in the appendiceal wall (Fig. 5b, Additional file 3: video S3). Based on these findings, she was diagnosed with de Garengeot hernia. Antibiotics (levofloxacin 500 mg/day) were administered to prevent potential development of appendicitis, and an elective surgery was performed seven days later.

**Fig. 6:**
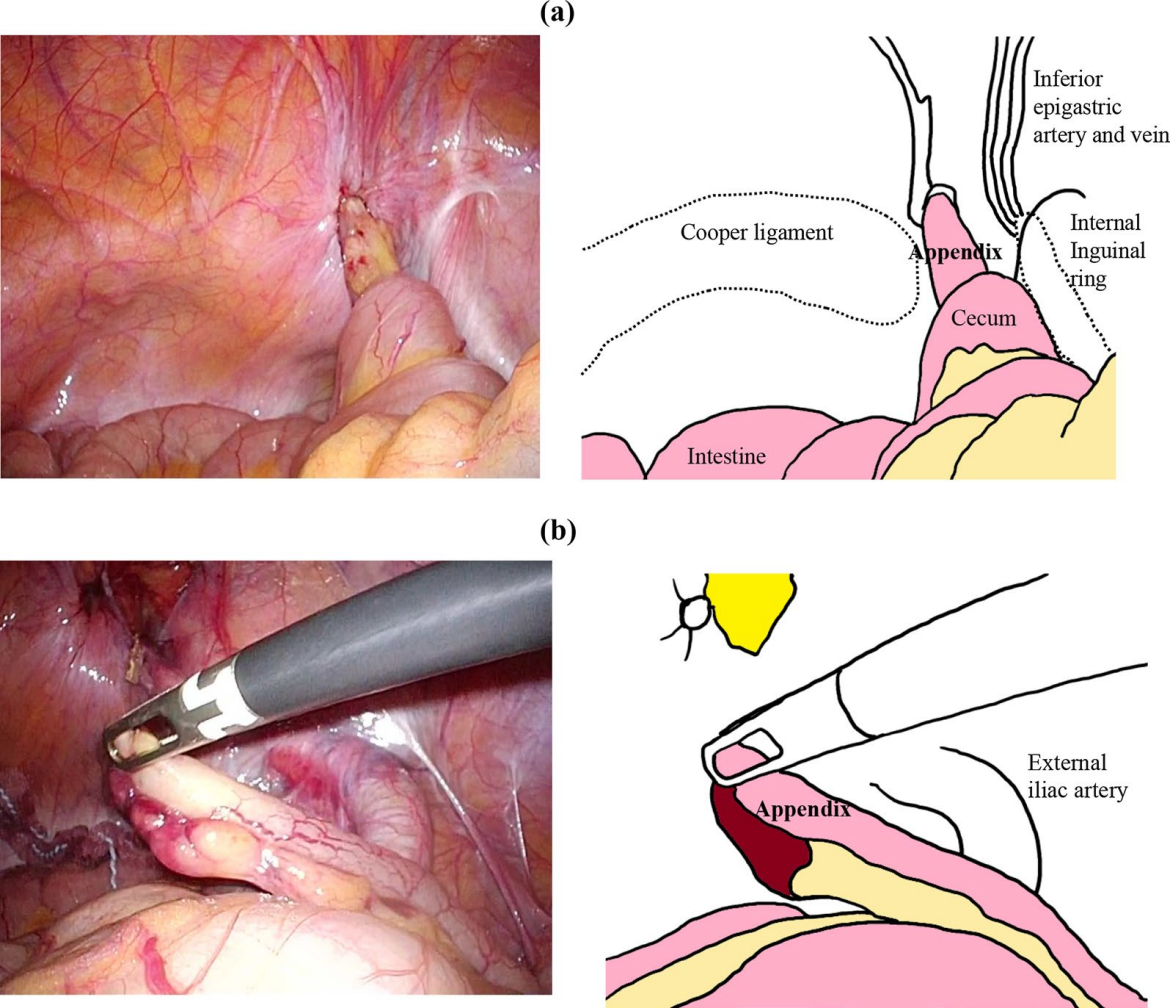
Laparoscopic image (Case 2). **a** The appendiceal tip was incarcerated in the right femoral ring. **b** The appendiceal tip was restored to the free abdominal cavity during ablation of the preperitoneal space. No enlargement, congestion, or color changes were noted in the appendix

The patient was placed in the supine position under general anesthesia, and one 5-mm port each was placed on the umbilicus, umbilical level of the right side of the abdomen, and left lower abdomen. Laparoscopy revealed an incarcerated appendiceal tip in the right femoral ring, which was not reducible by traction (Fig. 6a, Additional file 4: video S4). The peritoneum was incised, and parietalization performed. The appendiceal tip was restored to the free abdominal cavity during ablation of the preperitoneal space. Because there was no enlargement, congestion, or color change in the appendix (Additional file 5: video S5), appendicectomy was not performed. Versatex mesh 14 cm × 9 cm (Covidien) was placed in the preperitoneal space and fixed with an Absorber Tack 5 mm (Covidien). The peritoneum was closed with continuous suturing using a 3–0 Polysorb (Covidien).

The postoperative course was uneventful, and she has no signs of hernia recurrence or appendicitis 5 months postoperatively.

## Discussion

Because de Garengeot hernia is a rare disease, it is often difficult to diagnose preoperatively. In addition, the disease is frequently associated with acute appendicitis, necessitating emergent herniorrhaphy and appendectomy [8–11]. But emergency surgery can impose social and psychological burden on patients. In the two abovementioned cases, we performed elective herniorrhaphy without appendectomy because the clinical US and intraoperative findings did not indicate significant inflammation or circulatory compromise in the appendix.

To the best of our knowledge, there have been nine cases of de Garengeot hernia wherein appendix-preserving herniorrhaphy was performed in English and Japanese literature (Table 1) [10, 12–14]. The median age was 76 years (range: 70–78 years), and all patients were female. At presentation, the median white blood cell count and CRP level were 4520/μL (range: 3400–9500) and 0.04 mg/dL (range: 0.02–2.23), respectively. Most cases were diagnosed as de Garengeot hernia using CT, and US was performed in three cases (including ours). In our two cases, B-mode US showed a clear appendiceal wall structure and no enlargement of the appendix, and color Doppler US showed pulsatile blood flow signals in the appendiceal wall, suggesting the absence of inflammation or circulatory compromise. In case 2, we anticipated the development of appendicitis because of the slightly elevated WBC and CRP levels, and therefore administered antibiotics. Elective surgery was performed 28 and seven days later in case 1 and 2, respectively. Among the nine cases with appendix-preserving herniorrhaphy, the anterior approach was used in seven cases, and TAPP in our two cases. The appendix was preserved on the basis of laparoscopic findings.

**Table 1:** Reported cases of appendix preserving herniorrhaphy for de Garengeot hernia in English and Japanese literature

No.	Year	Author	Country	Number of patients	Age	Sex	WBC count (/μl)	CRP (mg/dL)	Diagnosis of de Garengeot hernia by CT	US findings	Preoperative diagnosis of appendicitis	Duration between diagnosis and surgery	Intraoperative findings of the appendix	Surgical method
1 ~ 4	2007	Sharma [10]	UK	4	nd	nd	nd	nd	nd	nd	Not diagnosed	< 10 h	Normal appendix	A
5	2014	Mizuno [12]	Japan	1	70	Female	5000	0.04	( +)	Not performed	No	nd	Very slight inflammation of appendix	A
6	2016	Jin [13]	UK	1	78	Female	WNL	WNL	( +)	A cystic mass and bowel loop within the hernia sac	Incarcerated appendix	Emergency surgery	A healthy appendix	A
7	2020	Uchida [14]	Japan	1	78	Female	4040	Negative	( +)	Not performed	nd	nd	No inflammation	A
8	–	Case1	Japan	1	76	Female	3400	0.02	( +)	Normal diameter, and clear wall structure of the appendix. Color doppler showed pulsatile blood flow signals in the appendiceal wall	No	28 days	Gomes: Grade 1 Guenter: Class1	TAPP
9	–	Case2	Japan	1	70	Female	9500	2.23	( +)	Same as above	No	7 days	Gomes: Grade0 Guenter: Class1	TAPP

*A* anterior approach, *TAPP* transabdominal preperitoneal approach, *nd* not described, *WNL* within normal limit

US and CT are highly useful because they can easily obtain tomographic images of the entire appendix. US is different from CT because it has a high spatial resolution and real-time capability, and can obtain tomographic images in any axis and evaluate vascularity using the color Doppler method although it depends on sonographers’ skill and experience. The US criteria for appendicitis include (1) enlargement of the appendix (> 6 mm), (2) maximum tenderness with a probe just above the appendix, (3) appendiceal wall thickness (≥ 3 mm), (4) loss of wall structure, (5) hyperechoic periappendiceal tissue, (6) periappendiceal fluid retention, (7) appendicolith, and (8) hypervascularity (early stage) or avascularity (necrotic stage) of the appendiceal wall [15, 16] (Table 2). The presence of complex periappendiceal fluid, as well as greater maximum appendiceal diameter and the presence of an appendicolith are significantly associated with perforation [17]. In this report, the US findings included an unswollen appendix, clear wall structure, and pulsatile blood signals, which suggested the absence of acute appendicitis and circulatory compromise.

**Table 2:** US signs of acute appendicitis [16]

Direct signs	Indirect signs
Non-compressibility of the appendix	Free fluid surrounding appendix
Perforation: appendix might be compressible	Local abscess formation
Diameter of the appendix > 6 mm	Increased echogenicity of local mesenteric fat
Single wall thickness ≥ 3 mm	Enlarged local mesenteric lymph nodes
Unclear wall structure	Thickening of the peritoneum
Hypoechoic fluid-filled lumen	Secondary small bowel obstruction
Hyperechoic mucosa/submucosa	
Hypoechoic muscularis layer	
Appendicolith: hyperechoic with posterior shadowing	
Colour Doppler and contrast-enhanced US:	
Hypervascularity in early stages of acute appendicitis	
Hypo- to avascularity in abscess and necrosis	

The indication for appendectomy during surgery for de Garengeot hernia should be determined based on intraoperative findings. Gomes et al. [18] and Guenther et al. [19] classified the severity of appendicitis based on intraoperative gross findings in the appendix (Tables 3 and 4). Case 1 and 2 corresponded to Grades 1 and 0 of the Gomes classification and Class 1 of the Guenther classification, respectively.

**Table 3:** Laparoscopic grading system of acute appendicitis based on the gross findings of the appendix proposed by Gomes et al. [18]

Grade	Laparoscopic findings
Grade 0	Normal looking appendix
Grade 1	Hyperemia and edema
Grade 2	Fibrinous exudate
Grade 3A	Segmental necrosis
Grade 3B	Base necrosis
Grade 4A	Abscess
Grade 4B	Regional peritonitis
Grade 5	Difuse peritonitis

**Table 4:** Classification of De Garengeot Hernia according to the gross appearance of the appendix proposed by Guenter et al. [19]

Class	Description
Class 1	Normal appearing appendix
Class 2	
2A	Erythematous, inflamed, or congested appendix
2B	Erythematous, inflamed, or congested appendix
	AND
	Erythema of the cecum or other segment of large or small intestine
Class 3	
3A	Necrosis of the appendix, isolated to the tip
3B	Necrosis of the appendix, involving the entire appendix
Class 4	Necrosis of the appendix
	AND
	Necrosis of the cecum or other segment of large or small intestine
Class 5	Perforated appendix, abscess, or fistula

There is a risk of mesh infection when inflamed appendicitis is resected. After confirming that there is no appendiceal inflammation by laparoscopy, herniorrhaphy with mesh makes the surgery safer. Because the appendix is important to produce IgA and regulation of intestinal microflora [20, 21, 21], unnecessary appendectomy should be avoided.

When de Garengeot hernia is diagnosed, precise evaluation of inflammation and circulatory compromise in the appendix allows determination of the level of surgical emergency (emergency/elective). If the intraoperative findings do not show appendicitis or circulatory compromise, the appendix can be preserved.

## Supplementary Information

**Additional file 1: Video S1.** Color Doppler US showed pulsatile blood flow signals in the appendiceal wall.

**Additional file 2: Video S2.** Laparoscopy showed incarceration of the median umbilical fold into the right femoral ring and the free appendix in the abdominal cavity. A fibrous band was also found between the right femoral ring and appendiceal tip, suggesting that the appendiceal tip had previously been in the femoral ring.

**Additional file 3: Video S3.** Color Doppler US showed pulsatile blood flow signals in the appendiceal wall.

**Additional file 4: Video S4.** Laparoscopy revealed an incarcerated appendiceal tip in the right femoral ring, which was not reducible by traction.

**Additional file 5: Video S5.** The appendiceal tip was restored to the free abdominal cavity during ablation of the preperitoneal space. There was no enlargement, congestion, or color change in the appendix.

## Data Availability

Data sharing is not applicable to this article as no datasets were generated or analysed during the current study.

## Abbreviations

CRP: C-reactive protein
CT: Computed tomography
TAPP: Transabdominal preperitoneal approach
US: Ultrasonography
WBC: White blood cell
